# Tracking the Evolution of Polymerase Genes of Influenza A Viruses during Interspecies Transmission between Avian and Swine Hosts

**DOI:** 10.3389/fmicb.2016.02118

**Published:** 2016-12-26

**Authors:** Nipawit Karnbunchob, Ryosuke Omori, Heidi L. Tessmer, Kimihito Ito

**Affiliations:** ^1^Division of Bioinformatics, Research Center for Zoonosis Control, Hokkaido UniversitySapporo, Japan; ^2^Precursory Research for Embryonic Science and Technology, Japan Science and Technology AgencyKawaguchi, Japan

**Keywords:** influenza A viruses, polymerase complex, host range, interspecies transmission, reciprocal best hits

## Abstract

Human influenza pandemics have historically been caused by reassortant influenza A viruses using genes from human and avian viruses. This genetic reassortment between human and avian viruses has been known to occur in swine during viral circulation, as swine are capable of circulating both avian and human viruses. Therefore, avian-to-swine transmission of viruses plays an important role in the emergence of new pandemic strains. The amino acids at several positions on PB2, PB1, and PA are known to determine the host range of influenza A viruses. In this paper, we track viral transmission between avian and swine to investigate the evolution on polymerase genes associated with their hosts. We traced viral transmissions between avian and swine hosts by using nucleotide sequences of avian viruses and swine viruses registered in the NCBI GenBank. Using BLAST and the reciprocal best hits technique, we found 32, 33, and 30 pairs of avian and swine nucleotide sequences that may be associated with avian-to-swine transmissions for PB2, PB1, and PA genes, respectively. Then, we examined the amino acid substitutions involved in these sporadic transmissions. On average, avian-to-swine transmission pairs had 5.47, 3.73, and 5.13 amino acid substitutions on PB2, PB1, and PA, respectively. However, amino acid substitutions were distributed over the positions, and few positions showed common substitutions in the multiple transmission events. Statistical tests on the number of repeated amino acid substitutions suggested that no specific positions on PB2 and PA may be required for avian viruses to infect swine. We also found that avian viruses that transmitted to swine tend to process I478V substitutions on PB2 before interspecies transmission events. Furthermore, most mutations occurred after the interspecies transmissions, possibly due to selective viral adaptation to swine.

## Introduction

The influenza A virus is a negative-sense single-stranded RNA virus that infects humans as well as a wide range of animals (Webster et al., 1992; Kuiken et al., 2004; Tong et al., 2012). Wild aquatic birds, such as wild ducks, geese, gulls, and shorebirds, are the natural reservoirs of the influenza A virus (Kida et al., 1988; Webster et al., 1992). Human influenza pandemics have historically been caused by genetic reassortment of human and avian influenza A viruses, and this reassortment typically occurs among viruses circulating in swine (Webster and Laver, 1972; Scholtissek et al., 1978; Kawaoka et al., 1989; Yasuda et al., 1991; Smith et al., 2009). Experimental studies have suggested that swine are susceptible to both human (Kundin, 1970) and avian viruses (Kida et al., 1994). Thus, the avian-to-swine transmission of influenza A viruses is an important factor contributing to the emergence of new pandemic strains.

Influenza A viruses are composed of eight gene segments, which encode at least 17 viral proteins (Dubois et al., 2014). Of these, the polymerase complex consisting of PB2, PB1, and PA is responsible for viral replication in host cells. The PB2 protein is responsible for the cap binding of host’s mRNA (Webster et al., 1992). The PB1 protein is associated with the catalytic activity of RNA synthesis (Kobayashi et al., 1996; Neumann et al., 2004; Elton et al., 2006). The PA protein is involved in endonuclease activity of the polymerase complex for RNA replication (Dias et al., 2009; Yuan et al., 2009).

The amino acids at several positions on the polymerase complex have been known to determine the host range of influenza A viruses. The amino acid substitution from Glutamic acid (E) to Lysine (K) at position 627 on PB2 of avian viruses increases viral replication in mammalian hosts (Subbarao et al., 1993; Hatta et al., 2001; Shinya et al., 2004; Mok et al., 2014). Two simultaneous amino acid mutations from Valine (V) to Serine (S) at position 715 and from Isoleucine (I) to Serine (S) at position 750 in PB1 are known to reduce the number of cRNA and mRNA (Sugiyama et al., 2009). Several amino acid substitutions in PA were reported to affect viral replication in mammals (Yamayoshi et al., 2014). Most of these studies discuss mammalian adaptation of avian viruses using mouse models of influenza infections. Currently, there is little information about the viral adaptation of avian viruses to swine.

It is important to know which amino acid substitutions on the polymerase complex determine the host range of avian influenza A viruses. A typical alignment-based approach compares consensus sequences of avian viruses and viruses isolated from other hosts, and the different amino acids in their alignments are considered as signature residues for each host (Chen et al., 2006). However, the alignment-based approach is known to be unable to distinguish the founder effect from selective viral adaptation (Tamuri et al., 2009). In order to clarify which amino acid substitutions on viral polymerase are beneficial for avian viruses to transmit to swine, we need to develop a new approach to finding important amino acid substitutions, and each substitution needs to be assessed by statistical tests.

The reciprocal best hits method has been widely used to identify orthologous genes, which are genes shared by different organisms (Tatusov et al., 1997; Bork and Koonin, 1998; Moreno-Hagalsieb and Latimer, 2008). Given two sets of sequences, *X* and *Y*, a pair of sequences *x* in *X* and *y* in *Y* is called a reciprocal best hit, if *x* is the most similar sequence among *X* to *y* and *y* is the most similar sequence among *Y* to *x*. Using a homology search program, such as BLAST, one can retrieve avian virus sequences similar to swine virus sequences. However, if a database contains more than one sequence similar to a sequence associated with a transmission event, simple BLAST searches using a threshold may give multiple combinations of similar sequences. By applying the reciprocal best hits method to the nucleotide sequences of viruses isolated from avian and swine, we can identify pairs of viruses associated with interspecies transmissions without double counting.

In this paper, we investigate the evolution of polymerase genes of influenza A viruses during viral transmission from avian to swine. A pair of nearly identical nucleotide sequences, one of which is from avian viruses and the other from swine, can be considered a footprint of viral transmission between avian and swine hosts. We denote such a pair as a transmission pair. By using BLAST and the reciprocal best hits technique, we explore transmission pairs associated with sporadic transmissions of avian viruses to swine. By analyzing the number of amino acid substitutions on the polymerase proteins found in the transmission pairs of polymerase genes between avian and swine viruses, we examine whether or not these amino acid substitutions are important for interspecies transmission of influenza A viruses between avian and swine hosts.

## Materials and Methods

### Nucleotide Sequences

The nucleotide sequences of PB2, PB1, and PA genes of avian and swine influenza A viruses were downloaded from the National Center for Biotechnology Information (NCBI) Influenza Virus Resource (Bao et al., 2008). Identical nucleotide sequences were removed using the collapse option of the database. Nucleotide sequences containing ambiguous nucleotides or which were less than 95% of the full-length gene were excluded. We obtained 7408, 7531, and 7576 nucleotide sequences of PB2, PB1, and PA genes of influenza A viruses isolated from avian hosts, and 1283, 1340, and 1304 nucleotide sequences of PB2, PB1, and PA genes of influenza A viruses isolated from swine (**Table 1**). We downloaded all the available nucleotide sequences on August 25, 2013.

**Table 1:** The number of nucleotide sequences of PB2, PB1, and PA genes used in this study.

Hosts	PB2	PB1	PA
Avian	7408	7531	7576
Swine	1283	1340	1304
Total	8691	8871	8880

### Bidirectional BLAST Searches between Avian and Swine Viruses

**FIGURE 1:**
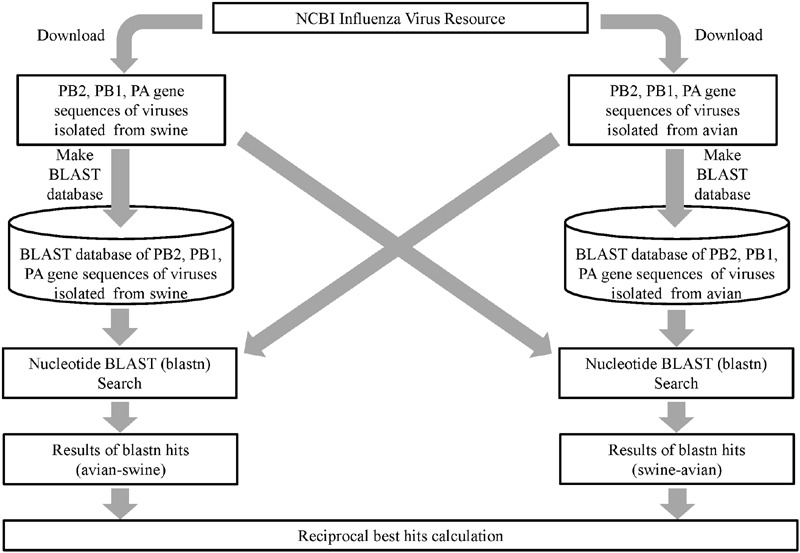
**A flow chart for bidirectional BLAST searches between avian and swine influenza A viruses.** The PB2, PB1, and PA gene sequences of viruses isolated from avian and swine were downloaded from the NCBI database. For each gene, two BLAST databases were constructed, one from the nucleotide sequences of avian isolates and the other from swine isolates. A blastn search was performed to search for similarities between the avian and swine virus isolates in two directions; first querying avian virus sequences against the swine virus sequence database and vice versa.

To identify similar nucleotide sequences between swine and avian viruses, we used Basic Local Alignment Search Tool (BLAST) (Altschul et al., 1990). For each of the polymerase gene segments, we constructed two BLAST databases – one for the nucleotide sequences of avian virus isolates and the other for those of swine virus isolates. BLAST homology searches were conducted bidirectionally using avian sequences as a query against swine sequences as subjects, and vice versa (**Figure 1**). The makeblastdb and blastn commands implemented in ncbi-blast-2.2.28+ were used to construct the databases and to conduct the homology search (Altschul et al., 1990).

### Tracking Transmissions by Reciprocal Best Hits Technique

To track the interspecies transmissions of influenza A viruses between avian and swine hosts, we explored avian and swine virus polymerase sequence pairs that are similar to each other by using the reciprocal best hits method. We consider a pair of avian and swine virus sequences that are similar to each other as a footprint of viral transmission between avian and swine hosts, and we call such a pair a transmission pair.

Given two sets of sequences *X* = {*x*_1_, *x*_2_, *x*_3_,…, *x_m_*} and *Y* = {*y*_1_, *y*_2_, *y*_3_,…, *y_n_*}, reciprocal best hit pairs can be found as follows: First, for each *x_i_* in *X*, we perform a BLAST search using *x_i_* against *Y* and record its top hit as Top(*x_i_*). Second, for each *y_j_* in *Y*, we perform a BLAST search using *y_j_* against *X* and record its top hit as Top(*y_j_*). Finally, all the pairs of (*x_i_, y_j_*) that satisfy Top(*x_i_*) = *y_j_* and Top(*y_j_*) = *x_i_* are output as reciprocal best hits.

**FIGURE 2:**
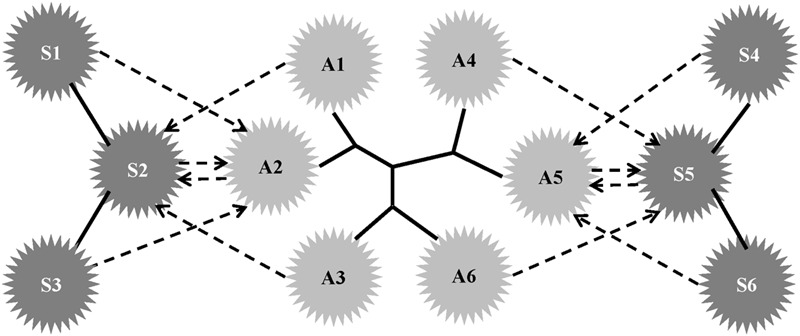
**Tracking transmissions by reciprocal best hits.** A1–A6 represent viruses isolated from avian hosts. S1–S6 represent viruses isolated from swine hosts. Solid lines represent phylogenetic relationships. Dashed arrows represent the top hits found by a blastn search. A pair of sequences, each of which is the top hit from the other, is called a reciprocal best hit. Pairs (A2, S2) and (A5, S5) are reciprocal best BLAST hits, and we assume such a pair is associated with an interspecies transmission event between avian and swine.

**Figure 2** illustrates how the reciprocal best hits method can track viral transmission events between avian and swine. A1–A6 represent viruses isolated from avian hosts, and S1–S6 represent viruses isolated from swine hosts. Solid lines represent phylogenetic relationships. Dashed arrows represent the top hits found by a blastn search. Pairs (A2, S2) and (A5, S5) are reciprocal best BLAST hits.

A pair of nucleotide sequences found to be reciprocal best hits and having more than 95% identity with an *E*-value of zero were selected and determined as a transmission pair between avian and swine. We used a custom-made Python program to find reciprocal best hits from BLAST results files. The program is available upon request.

### Determination of Transmission Direction by Phylogenetic Analysis

To determine the direction of interspecies transmission, we constructed a phylogenetic tree for each polymerase gene segment. For each polymerase gene, nucleotide sequences of both avian isolates and swine isolates were aligned using Multiple Alignment with Fast Fourier Transform (MAFFT) version 7.245 (Katoh and Standley, 2013). Phylogenetic trees of avian and swine isolates were constructed using the neighbor-joining method (Saitou and Nei, 1987) with ClustalX version 2.1 (Larkin et al., 2007). We used Dendroscope version 3.4.1 (Huson and Scornavacca, 2012) to visualize transmission pairs in phylogenetic trees. A transmission pair found in an avian virus clade was considered an avian-to-swine transmission. In contrast, a transmission pair found in a swine virus clade was considered a possible case of swine-to-avian transmission.

### Analysis of Amino Acid Substitutions

To analyze the tendencies in amino acid substitutions in polymerase during avian-to-swine transmission, nucleotide sequences of the transmission pairs of PB2, PB1, and PA genes were translated to protein sequences. For each transmission pair in the avian-to-swine direction, the protein sequence of the avian virus and swine virus were compared, and the amino acid substitutions identified. By the nature of the sequences registered in the database, the positions at the beginning and end were lacking nucleotide information. Gaps found at the beginning and end in transmission pairs were excluded from analysis, and gaps in the other regions were counted in the same way as substitutions.

### Statistical Analysis on the Number of Amino Acid Substitutions

If an amino acid position on a polymerase protein determines the host range of viruses, then such a position should be substituted into different amino acids at interspecies transmission events. To determine whether or not some amino acid positions are important for interspecies transmission, we set our null hypothesis to “amino acid substitutions randomly occurred over all positions.” We first estimated how many times amino acid substitutions can naturally occur at the same position with random substitutions at independent transmission events.

Let *m* be the total number of amino acid substitutions occurring on a protein sequence of length *l* at independent transmission events. Considering multiple transmission events, the total number of amino acid substitutions, *m*, may exceed the sequence length, *l*, when we have a large number of transmission events. By assuming amino acid substitutions occur equally over all the positions, the probability that at least one amino acid position is substituted more than *n* times can be calculated by the following formula:

(1) $$p=1-{\left(\sum_{k=0}^{n}\left(\begin{array}{c} m\\ k \end{array}\right){\left(\frac{1}{l}\right)}^{\text{k}}{\left(1-\left(\frac{1}{l}\right)\right)}^{m-k}\right)}^{l}$$

If the probability for the maximum number of amino acid substitutions in the observed data is smaller than the significance level (*p* < 0.05), we can reject the null hypothesis and conclude that some positions tend to be substituted more frequently than other positions. We confirmed the validity of the formula by comparing it with the multiple substitution probability obtained from Monte Carlo simulations.

### Statistical Analysis of Amino Acid Substitutions before and after Avian-to-Swine Transmissions

To characterize the genetic background of avian influenza A viruses that were able to infect swine, we compared consensus amino acid sequences of PB2, PB1, and PA of avian influenza A viruses found in avian-to-swine transmission pairs against consensus amino acids of all avian viruses. Similarly, to characterize the viral adaptation after interspecies transmission from avian to swine, we compared consensus amino acid sequences of PB2, PB1, and PA of swine influenza A viruses found in avian-to-swine transmission pairs against consensus amino acid sequences of all swine viruses. For each position having different consensus amino acids between all avian viruses and avian isolates in avian-to-swine transmission pairs, amino acid variations were further analyzed. We set our null hypothesis to “amino acid compositions at a given position in the two alignments are derived from the same distribution.” We use Fisher’s exact test (Fisher, 1922) to calculate the probability that the amino acid counts in two alignments come from the same distribution. If this *p*-value is smaller than the significance level, then the null hypothesis will be rejected.

## Results

### Transmission Pairs Found in Reciprocal Best Hits

To track the interspecies transmission of influenza A viruses between avian and swine hosts, we looked for nearly identical avian and swine virus polymerase sequences. The reciprocal best hits method found 41, 45, and 45 pairs of avian and swine sequences for PB2, PB1, and PA genes, respectively. All of the reciprocal best hits pairs on the PB2, PB1, and PA genes showed a BLAST *E*-value of zero. Of these reciprocal best hits pairs, 41 pairs for PB2, 44 pairs for PB1, and 42 pairs for PA had more than 95% identity (Supplementary Tables 1–3). We considered these nearly identical pairs as transmission pairs, which would be associated with transmission of avian viruses to swine or transmission of swine viruses to avian.

The transmission pairs between avian and swine sequences suggested that interspecies transmissions occurred frequently at adjacent places and their isolation years were close to each other. Of 41 transmission pairs for PB2, 32 pairs (78%) were from the same country and 32 pairs (78%) were isolated within 3 years of one another (Supplementary Table 1). Of 44 transmission pairs for PB1, 34 pairs (77%) were from the same country and 33 pairs (75%) were isolated within 3 years (Supplementary Table 2). Of 42 transmission pairs for PA, 33 pairs (79%) were from the same country and 31 pairs (74%) were isolated within 3 years (Supplementary Table 3). Although there are a few exceptions, these results suggest that transmission occurred between avian and swine located in adjacent areas.

### Direction of the Transmission between Avian and Swine

The clade distribution of transmission pairs in phylogenetic trees showed similar trends among PB2, PB1, and PA genes (Supplementary Figure 1). Out of 41 transmission pairs of PB2, 32 (78%) were found in avian clades and 8 (20%) were found in swine clades (Supplementary Table 1). Out of 44 transmission pairs of PB1, 33 (75%) were found in avian clades and 10 (23%) were found in swine clades (Supplementary Table 2). Out of 42 transmission pairs of PA, 30 (71%) were found in avian clades and 11 (26%) were found in swine clades (Supplementary Table 3). A transmission pair found in an avian clade can be considered an avian-to-swine transmission and vice versa. We did not determine the transmission direction for a pair in which one sequence is in an avian clade and the other in a swine clade. In some avian-to-swine transmission pairs, swine viruses were isolated before avian viruses. Similar contrary cases were also observed in swine-to-avian transmissions. The polymerase complex of influenza A viruses is known to evolve slowly because of functional constraints on protein evolution (Gorman et al., 1990). The inconsistency between transmission direction and isolation order may be attributed to the slow evolution of polymerase complex and the delayed viral isolation from their source population. In summary, 78, 75, and 71% of transmission pairs could be associated with avian-to-swine transmission for PB2, PB1, and PA, respectively. In contrast, 20, 23, and 26% of transmission pairs could be swine-to-avian transmissions of PB2, PB1, and PA, respectively.

### Amino Acid Substitutions during Avian-to-Swine Transmissions

**FIGURE 3:**
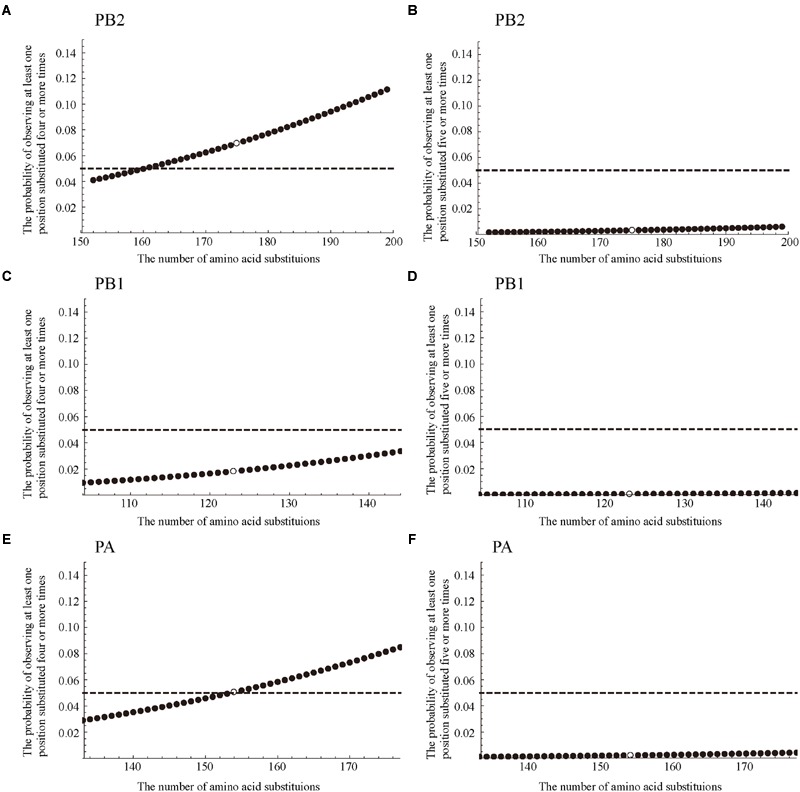
**The sensitivity of the significance on the position specific substitution count against the total number of substitutions.** White circles show the probability that at least one position is substituted more than given times under the number of substitutions observed in our dataset, and black circles show those of the 95% confidence intervals of the total number of substitutions. **(A,C,E)** show the probabilities of observing at least one position substituted four or more times for PB2, PB1, and PA, respectively. **(B,D,F)** show the probabilities of observing at least one position substituted five or more times for PB2, PB1, and PA, respectively.

The PB2 protein is 759 amino acids long, and 175 amino acid substitutions were observed at 142 different positions on PB2 in the 32 avian-to-swine transmission pairs (**Table 2**). An avian-to-swine transmission pair of PB2 has 5.47 amino acid substitutions on average. Note that the count for each position was weighted by the number of transmission pairs having an amino acid substitution at that position, i.e., (3 × 5)+(2 × 23)+(1 × 114) = 175, and this total count was averaged by the number of pairs, i.e., 175/32 = 5.47. When 175 substitutions were randomly distributed over 759 positions, the probability that we observed at least one position substituted four or more times is 0.070 (**Figure 3A**), and the probability that we observed at least one position substituted five or more times is 0.0032 (**Figure 3B**), according to formula (1). To reject the random substitution null hypothesis, we need at least five amino acid substitutions at the same position on the PB2 protein. Among 759 positions on PB2, no position was substituted four or more times. The observed number of multiple amino acid substitutions at the same positions on the PB2 protein was not statistically significant to reject the null hypothesis with a significance level of 0.05. Therefore, we cannot say that avian viruses require amino acid substitutions on specific positions of PB2 to infect swine.

**Table 2:** Positions at which amino acid substitutions were observed on PB2 proteins in transmission pairs.

Position at PB2 protein	Number of amino acid substitutions^∗^	Number of positions
389, 473, 547, 570, 674	3	5
8, 129, 139, 147, 194, 255, 327, 338, 344, 354, 355, 446, 456, 461, 575, 613, 661, 667, 684, 699, 701, 756, 759	2	23
15, 23, 28, 51, 58, 64, 66, 67, 79, 81, 87, 89, 92, 95, 105, 106, 109, 113, 122, 130, 134, 136, 137, 142, 144, 152, 153, 157, 164, 168, 175, 177, 184, 186, 187, 188, 189, 191, 192, 199, 206, 221, 240, 253, 265, 274, 285, 292, 293, 295, 299, 300, 303, 307, 309, 312, 313, 315, 317, 318, 330, 336, 340, 343, 351, 352, 356, 365, 368, 374, 375, 376, 394, 395, 399, 416, 422, 433, 444, 451, 463, 464, 468, 480, 488, 503, 508, 509, 521, 526, 537, 543, 553, 574, 584, 588, 598, 618, 629, 648, 659, 663, 666, 678, 680, 702, 714, 715, 717, 723, 731, 732, 742, 748	1	114
Others	0	617
Total		759

*^∗^The number of amino acid substitutions is defined as the number of transmission pairs having amino acid substitutions at each position. The positions of avian–human signature residues identified by Chen et al. (2006) were underlined.*

The PB1 protein is 757 amino acids long, and we observed 123 amino acid substitutions at 105 different positions on PB1 in the 33 avian-to-swine transmission pairs (**Table 3**). An avian-to-swine transmission pair on PB1 has 3.73 amino acid substitutions on average. Here, the count for each position was weighted by the number of transmission pairs having an amino acid substitution at that position, i.e., (4 × 1)+(3 × 2)+(2 × 11)+(1 × 91) = 123, and this total count was averaged by the number of pairs, i.e., 123/33 = 3.73. When 123 substitutions were randomly distributed over 757 positions, the probability that we observed at least one position substituted four or more times is 0.018 (**Figure 3C**), according to formula (1). Among 757 positions on PB1, position 156 was substituted four times. The observed distribution of amino acid substitutions in the PB1 protein was statistically significant to reject the null hypothesis with a significance level of 0.05. However, these four the substitutions were Threonine (T) to Alanine (A), Threonine (T) to Lysine (K), Threonine (T) to Methionine (M), and Methionine (M) to Threonine (T); there was no clear pattern of amino acid substitutions. Furthermore, the consensus amino acid at this position was T in both avian and swine viruses, and the distribution of amino acids at position 156 in the transmission pairs showed no clear difference (*p* ≈ 1.0 with Fisher’s exact test, Supplementary Table 4). There are no research papers showing the importance of this position on the host range, as far as we know. It is difficult to understand a clear reason for such a number of amino acid substitutions at position 156.

**Table 3:** Positions at which amino acid substitutions were observed on PB1 proteins in transmission pairs.

Position at PB1 protein	Number of amino acid substitutions^∗^	Number of positions
156	4	1
375, 757	3	2
12, 113, 191, 213, 386, 430, 476, 567, 643, 645, 667	2	11
9, 11, 14, 18, 54, 55, 56, 57, 60, 75, 91, 106, 108, 114, 119, 125, 144, 155, 158, 166, 168, 174, 175, 179, 187, 192, 196, 200, 208, 209, 212, 230, 237, 239, 247, 254, 261, 268, 309, 315, 320, 327, 336, 346, 361, 364, 374, 383, 384, 388, 402, 419, 421, 433, 434, 448, 451, 454, 457, 458, 465, 509, 511, 516, 517, 521, 527, 563, 566, 569, 571, 577, 578, 587, 591, 595, 599, 640, 641, 670, 694, 696, 702, 717, 719, 730, 741, 746, 748, 753, 756	1	91
Others	0	652
Total		757

*^∗^The number of amino acid substitutions is defined as the number of transmission pairs having amino acid substitutions at each position. The positions of avian–human signature residues identified by Chen et al. (2006) were underlined.*

The PA protein is 716 amino acids long, and we observed 154 amino acid substitutions at 137 different positions on PA in the 30 avian-to-swine transmission pairs (**Table 4**). An avian-to-swine transmission pair of PA has 5.13 amino acid substitutions on average. Again, the count for each position was weighted by the number of transmission pairs having an amino acid substitution at that position, i.e., (3 × 1)+(2 × 15)+(1 × 121) = 154, and this total count was averaged by the number of pairs, i.e., 154/30 = 5.13. When 154 substitutions were randomly distributed over 716 positions, the probability that we observed at least one position substituted four or more times is 0.051 (**Figure 3E**), and the probability that we observed at least one position substituted five or more times is 0.0022 (**Figure 3F**), according to formula (1). To reject the random substitution null hypothesis, we need at least five amino acid substitutions at the same position on the PA protein. Among 716 positions on PA, no position was substituted four or more times. The observed distribution of amino acid substitutions in the PA protein was not statistically significant to reject the null hypothesis with a significance level of 0.05. Therefore, we cannot say that avian viruses require amino acid substitutions on specific positions of PA to infect swine.

**Table 4:** Positions at which amino acid substitutions were observed on PA proteins in transmission pairs.

Position at PA protein	Number of amino acid substitutions^∗^	Number of positions
441	3	1
57, 61, 80, 94, 186, 208, 231, 261, 272, 388, 554, 560, 585, 626, 684	2	15
3, 12, 14, 22, 27, 29, 38, 40, 78, 86, 88, 89, 90, 101, 109, 115, 118, 127, 135, 141, 158, 159, 169, 192, 201, 204, 210, 216, 224, 226, 238, 247, 252, 256, 262, 269, 277, 285, 308, 311, 312, 315, 316, 321, 323, 325, 335, 336, 345, 350, 353, 355, 361, 364, 372, 394, 403, 407, 413, 418, 424, 435, 437, 439, 440, 442, 450, 451, 469, 487, 490, 492, 496, 499, 501, 505, 512, 513, 518, 522, 528, 531, 535, 536, 538, 545, 547, 553, 558, 565, 566, 569, 570, 576, 580, 581, 587, 588, 589, 592, 595, 598, 602, 607, 615, 616, 617, 618, 619, 621, 625, 628, 665, 669, 680, 683, 693, 694, 696, 706, 715	1	121
Others	0	579
Total		716

*^∗^The number of amino acid substitutions is defined as the number of transmission pairs having amino acid substitutions at each position. The positions of avian–human signature residues identified by Chen et al. (2006) were underlined.*

### Analysis of Amino Acid Substitutions before and after Avian-to-Swine Transmissions

To characterize the genetic background of avian influenza A viruses that are able to infect swine, viral adaptation after interspecies transmission from avian to swine hosts was investigated. We compared consensus amino acid sequences of PB2, PB1, and PA for all avian isolates, avian and swine isolates in transmission pairs, and all swine isolates (**Table 5**). Nine positions on PB2, 13 positions on PB1, and five positions on PA had different consensus amino acids when compared to their consensus amino acid sequences. All the positions had the same consensus amino acids between avian and swine isolates on the avian-to-swine transmission pairs. All the positions, except 340 on PB2, had different amino acids between the consensus of swine isolates in transmission pairs and the consensus of all swine isolates, suggesting that positions, except 340 on PB2, were substituted during circulation in swine after avian-to-swine transmission. Amino acids at positions 65, 147, 271, 478, 588, 590, 591, and 645 on PB2, positions 179, 336, 339, 361, 375, 430, 486, 581, 584, 621, 638, 642, and 741 on PB1, and positions 362, 382, 388, 407, and 409 on PA appear to be substituted after interspecies transmission, possibly as a result of selective viral adaptation in swine.

**Table 5:** Comparison of consensus amino acids on PB2, PB1, and PA among avian isolates, avian and swine isolates in transmission pairs, and swine isolates.

Genes	Position	Consensus amino acid of all avian isolates	Consensus amino acid in avian-to-swine transmission pairs		Consensus amino acid of all swine isolates
			Avian isolates	Swine isolates	
PB2	65	E	E	E	D
	147	I	I	I	T
	271	T	T	T	A
	340	R	K	K	K
	478	I	V	V	I
	588	A	A	A	T
	590	G	G	G	S
	591	Q	Q	Q	R
	645	M	M	M	L
PB1	179	M	M	M	I
	336	V	V	V	I
	339	I	I	I	M
	361	S	S	S	R
	375	N	N	N	S
	430	R	R	R	K
	486	R	R	R	K
	581	E	E	E	D
	584	R	R	R	Q
	621	Q	Q	Q	R
	638	E	E	E	D
	642	N	N	N	S
	741	A	A	A	S
PA	362	K	K	K	R
	382	E	E	E	D
	388	S	S	S	G
	407	I	I	I	V
	409	S	S	S	N

The positions 340 and 478 on PB2 had different amino acids between the consensus of avian isolates in transmission pairs and the consensus of all avian isolates, suggesting that these positions were substituted before avian-to-swine transmission.

The amino acid at position 340 on PB2 of avian viruses in avian-to-swine transmission pairs tended to have Lysine (K), while Arginine (R) in this position is dominant in avian viruses. However, Fisher’s exact test on amino acid variation at position 340 showed *p* = 0.097 (**Table 6**), and we cannot reject our null hypothesis that avian PB2 sequences in the transmission pairs have the same amino acid composition at position 340 with other avian PB2 sequences.

**Table 6:** Variation of amino acids at position 340 on PB2 of avian viruses.

Amino acid at position 340	Number of sequences	
	Avian isolates excluding avian-to-swine transmission	Avian isolates in avian-to-swine transmission
R	4556	13
K	2797	19
S	12	0
G	8	0
E	2	0
N	1	0
Total	7376	32

*The probability that the observed frequencies of amino acids come from the same distribution is 0.097 using Fisher’s exact test.*

The amino acid at position 478 on PB2 of avian viruses in avian-to-swine transmission pairs tended to have Valine (V), while Isoleucine (I) in this position is dominant in avian viruses. Fisher’s exact test on amino acid variation at position 478 showed *p* = 6.1 × 10^-8^ (**Table 7**), indicating that avian PB2 sequences in the transmission pairs has a different amino acid composition at position 478 from other avian PB2 sequences. Therefore, I478V mutations on PB2 may be one of the most important amino acid substitutions for avian viruses to transmit to swine.

**Table 7:** Variation of amino acids at position 478 on PB2 of avian viruses.

Amino acid at position 478	Number of sequences	
	Avian isolates excluding avian-to-swine transmission	Avian isolates in avian-to-swine transmission
I	4194	2
V	3147	30
M	30	0
L	4	0
A	1	0
Total	7376	32

*The probability that the observed frequencies of amino acids come from the same distribution is 6.1 × 10^-8^ using Fisher’s exact test.*

## Discussion

Using BLAST and reciprocal best hits, we found 41, 44, and 42 transmission pairs between avian and swine hosts for PB2, PB1, and PA genes, respectively. These transmission pairs had more than 95% nucleotide identity, indicating that these pairs could be associated with interspecies transmission of influenza A viruses from avian to swine or swine to avian hosts. Phylogenetic analysis showed more than 70% of transmission pairs were associated with avian-to-swine transmissions. By comparing amino acid sequences of avian and swine isolates in the avian-to-swine transmission pairs, we examined amino acid substitutions during avian-to-swine transmissions. On average, avian-to-swine transmission pairs had 5.47, 3.73, and 5.13 amino acid substitutions on PB2, PB1, and PA, respectively. However, amino acid substitutions were distributed over the positions, and few positions showed common substitutions in the multiple transmission events. Statistical tests on the number of repeated amino acid substitutions suggested that no specific positions on PB2 and PA may be required for avian viruses to infect swine. We found that avian viruses involved in avian-to-swine transmissions tended to have Valine (V) at position 478 on PB2, while Isoleucine (I) at position 478 on PB2 are dominant in avian viruses. Statistical tests showed that the distribution of amino acids in avian viruses in avian-to-swine transmissions were different from that of all the avian viruses, suggesting that the I478V substitution may be beneficial for avian viruses to transmit to swine.

Our statistical test is based on the number of amino acid substitutions observed at the same position and the total number of amino acid substitutions at independent transmission events, which are *n* and *m* in formula (1), respectively. We assumed the substitution rates among all positions are equivalent with a point estimate of the observed substitution rates. In order to know how the point estimate affects the *p*-values of statistical tests, we assessed the significance using the 95% confidence intervals (CI) of the total number of amino acid substitutions. From the total number of amino acid substitutions observed in our dataset, the 95% CI of the total number were calculated as [153, 199], [104, 144], and [133, 177] for PB2, PB1, and PA, respectively using the binomial test. Substituting *m* in formula (1) with numbers in these ranges, we assessed the sensitivity of the significance on the position-specific count of amino acid substitutions to the total number of amino acid substitutions (**Figure 3**). PB2 and PA showed insignificant *p*-values (*p* ≥ 0.05), and we rejected our random null hypothesis for PB2 and PA. However, the significance varied with the total number of amino acid substitutions. PB2 showed insignificant *p*-values in a wide range of 95% CI on the total number of substitutions. In contrast, PA showed insignificant *p*-values in half of 95% CI, and the insignificance for PA may be attributed to sampling error. Further data collection is required to assess the significance of the position-specific count of the amino acid substitutions.

Among avian-to-swine transmission pairs of PB2, PB1, and PA genes, some swine viruses possessed different subtypes of HA from their corresponding avian viruses (Supplementary Tables 1–3). These viruses were reassortant viruses receiving HA genes of different subtypes before or after avian-to-swine transmissions. Since the HA protein is associated with receptor specificity in cell entry and is an important determinant of host range, the replacement of the HA subtype may affect amino acid substitutions on the polymerase complex. We examined the effect of HA replacement on the number of amino acid substitutions on PB2, PB1, and PA (Supplementary Tables 5–7). There was no significant difference between transmission pairs with and without HA replacement for PB2 and PB1 (*p* ≈ 1.0 for PB2 and PB1). However, transmission pairs of PA had significant differences in the number of amino acid substitutions between pairs having the same HA subtype versus different HA subtypes (*p* = 0.043). Transmission pairs with HA replacement had significantly larger numbers of amino acid substitutions compared to those without HA replacement. Supplementary Tables 8 and 9, respectively, show positions of amino acid substitutions on the avian-to-swine transmission pairs of PA with HA replacement and without HA replacement. The observed numbers of multiple amino acid substitutions at the same positions on the PA were not statistically significant to reject the null hypothesis, when the transmission pairs with and without HA replacement were analyzed separately (*p* ≥ 0.05).

**FIGURE 4:**
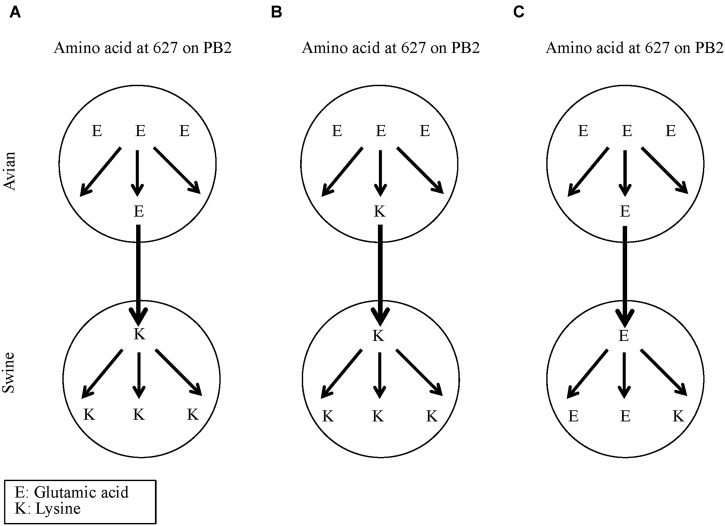
**The three possible hypotheses for the E627K substitution on the PB2 protein in the avian-to-swine transmission of influenza A viruses. (A)** E627K substitution occurred at transmission. **(B)** E627K substitution occurred before transmission. **(C)** E627K substitution occurred after transmission.

The glutamic acid (E) to lysine (K) substitution at position 627 (E627K) on PB2 is known to increase the replication ability of avian influenza viruses in mammalian hosts (Subbarao et al., 1993; Hatta et al., 2001; Shinya et al., 2004; Mok et al., 2014). We did not find this substitution in transmission pairs between avian and swine isolates. **Figure 4** shows three hypotheses that could explain this. Hypothesis A is that the amino acid change at position 627 occurred during the transmission from avian to swine hosts. However, we could not find any instance of this hypothesis. Hypothesis B is that the E627K amino acid change occurred before the transmission from avian to swine hosts, and hypothesis C is that the E627K amino acid changed after the transmission. All of the 32 avian-to-swine transmission pairs possessed E in both avian and swine. Therefore the E627K amino acid substitution on the PB2 protein is not necessary for avian influenza A viruses to infect swine (**Figure 4C**).

Avian viruses involved in avian-to-swine transmissions tended to have R340K and I478V substitutions on PB2. Both positions are known to be residues in the cap-binding domain of PB2 (Guilligay et al., 2008). Although the K at position 340 on PB2 is known to be associated with mammalian adaptation of avian viruses (Xiao et al., 2016), the Fisher’s exact test could not reject our null hypothesis. On the other hand, the Fisher’s exact test showed a significant difference in amino acid compositions at position 478 on PB2. The I478V substitution may be beneficial for avian viruses to transmit to swine. However, the dominant amino acid at position 478 on PB2 of avian viruses was I, that for avian-to-swine transmission pairs was V, and that for swine viruses was I again (**Table 5**), indicating that it does not determine the host range. It is unclear why position 478 tended to have V only during transmission. Our hypothesis is that the I478V substitution would be associated with a factor needed for swine to be infected with avian viruses in a natural setting. Experimental studies are needed to determine the effect of this mutation on the tissue tropism, viral growth, polymerase activity, protein expression, and pathogenicity.

Comparing amino acid sequences of influenza A viruses isolated from avian hosts and humans, Chen et al. (2006) identified amino acid positions as signature residues that may be required for avian viruses to infect humans. They have reported 8, 2, and 10 positions of signature residues on PB2, PB1, and PA respectively. Among these positions, amino acid substitutions at four positions (199, 588, 613, and 674) on PB2, two positions (327 and 336) on PB1, and one position (57) on PA were also found in the avian-to-swine transmission pairs in our study (**Tables 2–4**). However, our results suggested that amino acid substitutions at these positions may not be required for avian viruses to infect swine.

Phylogenetic analysis of transmission pairs in reciprocal best hits suggest that interspecies transmissions between avian and swine hosts occur in both directions. Several studies have reported the transmission from avian to swine hosts (Kida et al., 1988; Guan et al., 1996; Karasin et al., 2000; Ninomiya et al., 2002; Choi et al., 2005; Su et al., 2013) and our results on interspecies transmission from avian to swine are consistent with these studies. Experimental research has shown that most avian influenza A virus strains can infect swine (Kida et al., 1994). As described in the results section, avian influenza A viruses may not require specific amino acid substitutions in PB2 and PA to infect swine. Previous studies have also reported phylogenetic evidence of transmission from swine to avian (Olsen et al., 2003; Berhane et al., 2012). Only around 23% of transmission pairs in this study had a swine-to-avian direction. The difference in the number of avian-to-swine and swine-to-avian transmissions may be attributed to the high susceptibility of swine to avian viruses. Another factor that affects the imbalanced transmission direction is the difference in the prevalence of influenza A viruses in avian and swine hosts. The natural reservoirs of the influenza A virus are wild aquatic birds. The prevalence of influenza viruses in the mallard is more than 10% (Olsen et al., 2006), while the prevalence in swine is less than 5% (Corzo et al., 2013). The chance for a pig to be exposed to an avian virus is higher than the chance for a bird to be exposed to a swine virus. Since past pandemics of influenza have been caused by the viral transmission from avian to swine and then swine to human, our result highlights the importance of monitoring avian-to-swine transmission to reduce the chance of future influenza pandemics.

Our reciprocal best hits-based method is applicable to the transmission analysis of other host species or other infectious diseases. In this study, we focused on the interspecies transmission of influenza A viruses between avian and swine hosts. One important future research direction is to analyze transmission of influenza A viruses from avian hosts to other mammalian hosts, including humans, using our method. In our study, we found that avian viruses that transmitted to swine tend to process I478V substitutions on PB2 before interspecies transmission events. By analyzing amino acid substitutions on polymerase during avian-to-human transmissions of H5N1 and H7N9 influenza A viruses, we may be able to identify important amino acid substitutions for avian viruses to transmit to humans. One can also apply the same methodology to analyze the global trend of influenza transmission in humans (Russell et al., 2008). The methodology can also be applied to analyze the transmission of other pathogens, as long as we can access a large amount of their genomic data. Our strategy fully depends on the sequences registered in the NCBI database. To identify amino acid residues that determine the host range of a virus, we need to assess the importance of amino acid substitutions found in transmission pairs between different host species using a statistical test. If we do not have a sufficient amount of sequence information from a host species, the number of detectable transmission pairs becomes small, and it will be difficult to conduct a statistical test on amino acid substitutions. The greater the quantity of pathogens’ nucleotide sequences accumulated in public databases, the higher the chance to obtain meaningful results this method will have.

## Author Contributions

NK and KI conceived and designed the study. RO and KI designed the statistical analysis. NK analyzed the data. NK, HT, RO, and KI wrote the paper.

## Conflict of Interest Statement

The authors declare that the research was conducted in the absence of any commercial or financial relationships that could be construed as a potential conflict of interest.
