# Supplementary material for: Tracking the Evolution of Polymerase Genes of Influenza A Viruses during Interspecies Transmission between Avian and Swine Hosts
Source: Front Microbiol. 2016 Dec 26;7:2118. doi: 10.3389/fmicb.2016.02118 (PMC5183616; doi:10.3389/fmicb.2016.02118)
Supplement: Supplementary file 8 [file Table_8.DOCX]

**Table S8. Positions at which amino acid substitutions were observed on PA proteins in 23 transmission pairs with the same HA subtype**

| Position at PA protein | Number of amino acid substitutions* | Number of positions |
| --- | --- | --- |
| 61, 80, 94, 231, 261, 272, 684 | 2 | 7 |
| 12, 27, 38, 40, 57, 78, 86, 88, 89, 101, 115, 141, 158, 159, 186, 204, 208, 210, 224, 226, 256, 262, 269, 285, 311, 321, 323, 325, 353, 355, 361, 364, 388, 403, 407, 418, 437, 439, 440, 441, 450, 469, 492, 496, 499, 505, 513, 531, 535, 545, 547, 554, 560, 566, 576, 581, 585, 587, 588, 595, 598, 602, 607, 618, 621, 626, 680, 683, 693, 694, 696, 706, 715 | 1 | 73 |
| Others | 0 | 636 |
|  | **Total** | **716** |

**Note: ***The number of amino acid substitutions is defined as the number of transmission pairs having amino acid substitutions at each position. The positions of avian–human signature residues identified by Chen et al., 2006 have been underlined.
