# Supplementary material for: Tracking the Evolution of Polymerase Genes of Influenza A Viruses during Interspecies Transmission between Avian and Swine Hosts
Source: Front Microbiol. 2016 Dec 26;7:2118. doi: 10.3389/fmicb.2016.02118 (PMC5183616; doi:10.3389/fmicb.2016.02118)
Supplement: Supplementary file 9 [file Table_9.DOCX]

**Table S9. Positions at which amino acid substitutions were observed on PA proteins in 7 transmission pairs with different HA subtypes**

| Position at PA protein | Number of amino acid substitutions* | Number of positions |
| --- | --- | --- |
| 441  3, 14, 22, 29, 57, 90, 109, 118, 127, 135, 169, 186, 192, 201, 208, 216, 238, 247, 252, 277, 308, 312, 315, 316, 335, 336, 345, 350, 372, 388, 394, 413, 424, 435, 442, 451, 487, 490, 501, 512, 518, 522, 528, 536, 538, 553, 554, 558, 560, 565, 569, 570, 580, 585, 589, 592, 615, 616, 617, 619, 625, 626, 628, 665, 669 | 2  1 | 1  65 |
| Others | 0 | 650 |
|  | **Total** | **716** |

**Note: ***The number of amino acid substitutions is defined as the number of transmission pairs having amino acid substitutions at each position. The positions of avian–human signature residues identified by Chen et al., 2006 have been underlined.
