# Supplementary material for: Tracking the Evolution of Polymerase Genes of Influenza A Viruses during Interspecies Transmission between Avian and Swine Hosts
Source: Front Microbiol. 2016 Dec 26;7:2118. doi: 10.3389/fmicb.2016.02118 (PMC5183616; doi:10.3389/fmicb.2016.02118)
Supplement: Supplementary file 10 [file Presentation_1.PDF]

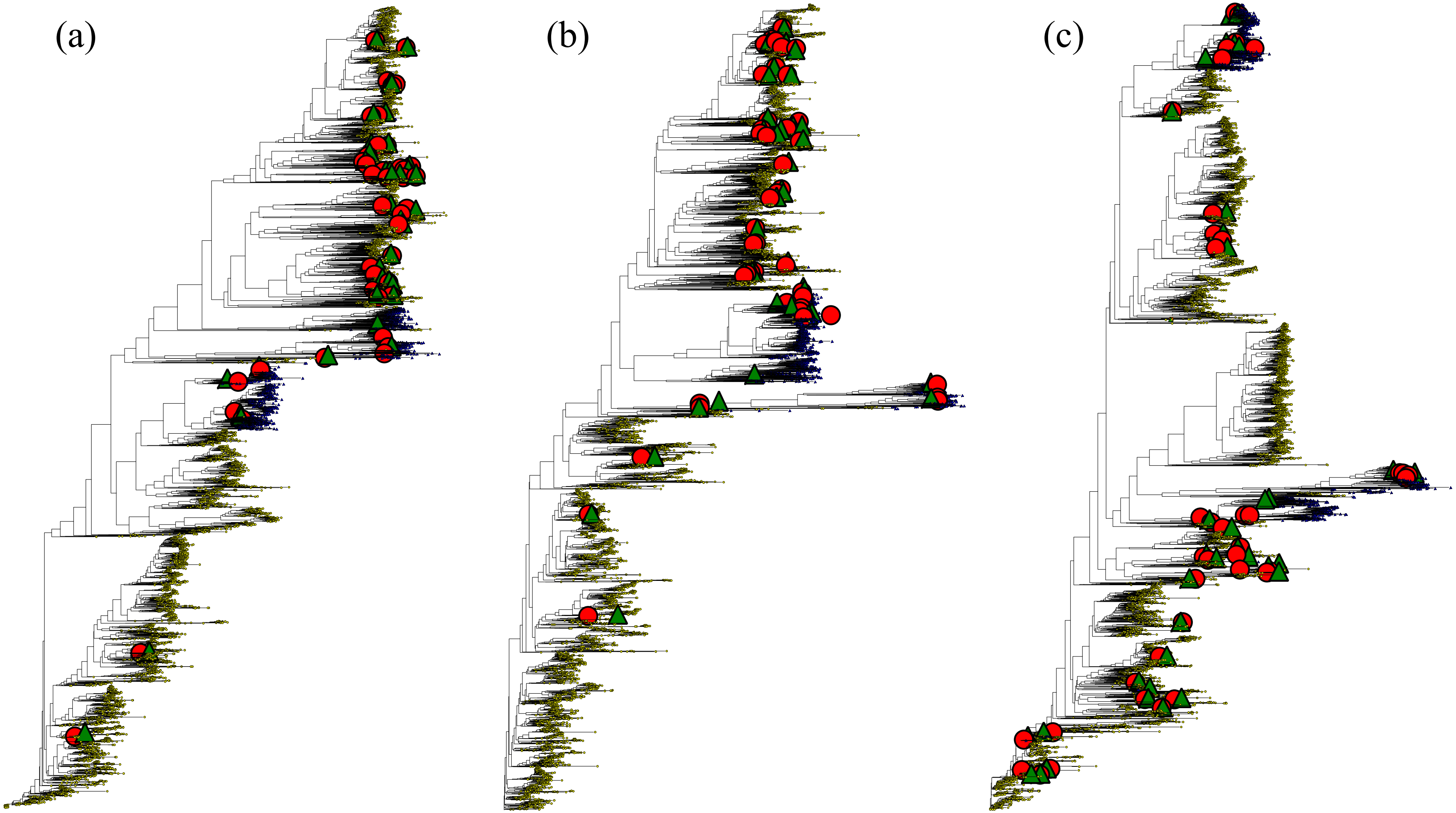

Figure S1. Phylogenetic trees of PB2 (a), PB1 (b), and PA (c) genes of avian and swine influenza A viruses. Small yellow circles represent sequences of avian viruses and small blue triangles represent sequences of swine viruses. Large red circles and green triangles represent avian and swine virus sequences, respectively, in reciprocal best hits pairs.
